# Thermally Stable, High Performance Transfer Doping of Diamond using Transition Metal Oxides

**DOI:** 10.1038/s41598-018-21579-4

**Published:** 2018-02-20

**Authors:** Kevin G. Crawford, Dongchen Qi, Jessica McGlynn, Tony G. Ivanov, Pankaj B. Shah, James Weil, Alexandre Tallaire, Alexey Y. Ganin, David A. J. Moran

**Affiliations:** 1 School of Engineering, University of Glasgow, Glasgow, G12 8LT United Kingdom; 2 Department of Chemistry and Physics, La Trobe Institute for Molecular Science, La Trobe University, Melbourne, Victoria, 3086 Australia; 3 School of Chemistry, University of Glasgow, Glasgow, G12 8LT United Kingdom; 4 Sensors and Electron Devices Directorate, U.S. Army Research Laboratory, Adelphi, Maryland 20783 USA; 5 LSPM-CNRS, Université Paris 13, Villetaneuse, 93430 France

## Abstract

We report on optimisation of the environmental stability and high temperature operation of surface transfer doping in hydrogen-terminated diamond using MoO_3_ and V_2_O_5_ surface acceptor layers*. In-situ* annealing of the hydrogenated diamond surface at 400 °C was found to be crucial to enhance long-term doping stability. High temperature sheet resistance measurements up to 300 °C were performed to examine doping thermal stability. Exposure of MoO_3_ and V_2_O_5_ transfer-doped hydrogen-terminated diamond samples up to a temperature of 300 °C in ambient air showed significant and irreversible loss in surface conductivity. Thermal stability was found to improve dramatically however when similar thermal treatment was performed in vacuum or in ambient air when the oxide layers were encapsulated with a protective layer of hydrogen silsesquioxane (HSQ). Inspection of the films by X-ray diffraction revealed greater crystallisation of the MoO_3_ layers following thermal treatment in ambient air compared to the V_2_O_5_ films which appeared to remain amorphous. These results suggest that proper encapsulation and passivation of these oxide materials as surface acceptor layers on hydrogen-terminated diamond is essential to maximise their environmental and thermal stability.

## Introduction

**Figure 1:**
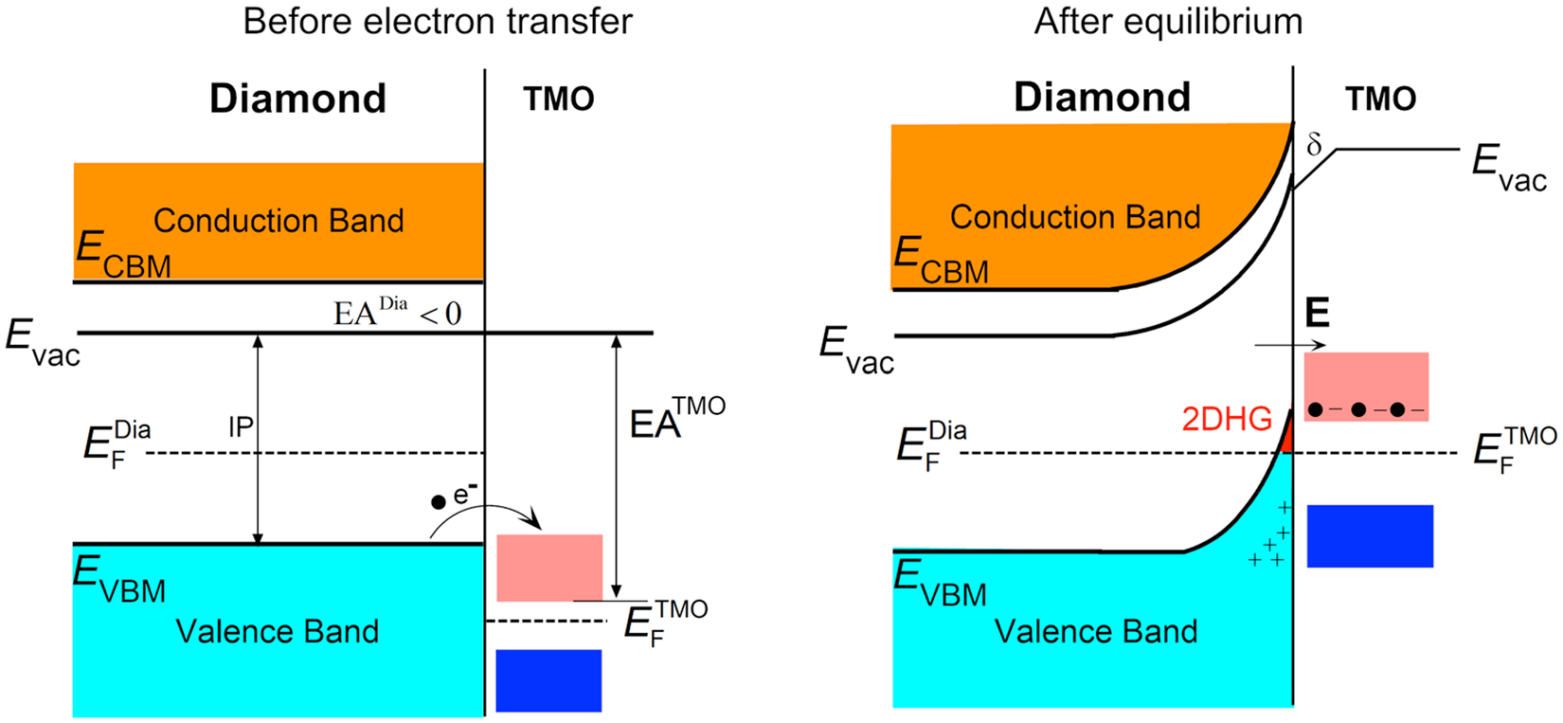
Band diagram illustrating the surface transfer doping mechanism of hydrogen terminated diamond by Transition Metal Oxides (TMO).

Due to its wide band-gap of 5.47 eV, extremely high thermal conductivity of >20 W/cm and intrinsically high breakdown field of 10 MV/cm diamond is very attractive for the production of high-power electronic devices such as field effect transistors (FET)^1–4^. Progress in the development of diamond-based electronic components however has been limited by the difficulties associated with doping^5^. The ability to introduce high densities of mobile charge in diamond through doping is essential for the realisation of high current-carrying, high power diamond-based electronic devices i.e. transistors and diodes. Boron has traditionally provided the most efficient substitutional doping technique to produce high conductivity p-type diamond^6^. Hole mobility is found to decrease substantially for high boron concentrations however and even for delta doping strategies which have recently been investigated^6–8^. Surface transfer doping of diamond offers an alternative to substitutional doping that alleviates the challenges of introducing impurity dopants into diamond’s tightly packed carbon lattice. Owing to the negative electron affinity produced by the hydrogen-terminated surface, diamond, when in intimate contact with a suitable molecular species, will form a sub-surface p-type channel. This “surface conductivity” occurs due to charge transfer of electrons from the diamond valence band to acceptor states provided by an acceptor material adjacent to the diamond surface, creating corresponding holes within the diamond and forming a 2-dimensional hole gas (2DHG)^9^. Historically, spontaneous accumulation of atmospheric adsorbents on the hydrogen-terminated diamond (H-diamond) surface when exposed to air have provided these surface acceptor states^1,9^. However, surface transfer doping of H-diamond when exposed to ambient air is highly sensitive to environmental conditions such as temperature, humidity and molecular composition^9^. Despite these limitations, promising electronic device performance has been demonstrated from air-exposed H-diamond. For example, air-exposed H-diamond FET devices have demonstrated drain current over 500 mA/mm^10^, a cut-off frequency (f_T_) of up to 53 GHz^11^ and a maximum frequency up to 120 GHz^12^. The reliance on air-borne acceptor species and sensitivity of the H-diamond surface to the ambient environment however, remains a significant limiting factor in the development of surface transfer-doped diamond electronic devices. To address these limitations, various work has investigated alternative surface acceptor materials on H-diamond^13^; this includes the fullerene molecule C_60_^14^, its fluorinated variants C_60_F_48_^15^, as well as F4-TCNQ^16^, each of which has successfully been shown to induce surface transfer doping of H-diamond. However, these materials still demonstrated limited temperature stability despite attempts to passivate some of them through encapsulation^17^. Another approach has demonstrated significantly improved stability up 550 °C in ambient atmosphere through atomic layer deposition (ALD) of Al_2_O_3_ onto H-diamond^18^. A similar approach used Al_2_O_3_ to encapsulate NO_2_ surface acceptor states on the H-diamond surface to achieve enhanced stability up to 200 °C in vacuum^19^. ALD deposition of HfO_2_ has also been examined for fabrication of H-diamond MOSFETs^20^. More recently, high electron affinity (EA) oxides such as Nb_2_O_5_, WO_3_, V_2_O_5_ and MoO_3_ have been shown to be highly effective for inducing surface transfer doping in H-diamond^21–24^. These oxides, when brought into contact with the H-diamond surface, induce the formation of a 2DHG below the diamond surface similar to that reported by exposure to ambient air. The process for transfer doping of H-diamond by transition metal oxide is illustrated in Fig. 1. Up to an order of magnitude increase in carrier concentration within the diamond has been reported using these oxide materials in comparison with that typically achieved through exposure to ambient air. Application of these oxides therefore presents a promising new strategy to further enhance the efficiency and high-power operation of planar diamond electronic devices. In addition to the associated increase in hole density, the potential gains in terms of stability and yield from use of these oxide layers are also of great interest for producing robust, real world diamond devices. Two of the most promising of these oxide materials for surface transfer doping of H-diamond are MoO_3_ and V_2_O_5_ which has consequently prompted exploration of their application in FET devices^25,26^. In this work, we explore the processing and resultant environmental and high-temperature stability of MoO_3_ and V_2_O_5_ films on H-diamond for high-performance transfer doping.

## Experimental

The experimental work in this study was undertaken using 4.5 × 4.5 mm optical grade single crystal type IIa [001] CVD diamond substrates provided by Element Six. Substrates were first cleaned in HNO_3_:HCl followed by H_2_SO_4_:HNO_3_ to remove any metallic and organic adsorbents. They were then treated with a microwave hydrogen plasma for 30 minutes at a temperature of 600 °C to terminate the surface of each with hydrogen. To allow for electrical characterisation by Hall measurement, van der Pauw (VDP) test structures were fabricated by applying silver paste to the corners of each of the hydrogenated samples. For the purpose of making such ‘large area’ ohmic contacts to H-diamond, silver contacts show a sufficiently low resistance and linear response^27^. Use of this VDP structure crucially minimises the risk of incurring contamination to or damage of the H-diamond surface using standard processing techniques *e.g*. exposure to resists, processing chemicals and electron beam. This method of VDP formation has also previously proven to be sufficiently accurate and robust for diamond substrates of these dimensions^21–23^. Following formation of the VDP contacts, each of the substrates then received varied processing to inspect the stability of the resultant transfer doping with time and at elevated temperatures. For ease of reference, Table 1 summarises the processing that each of these test substrates received. In summary: Substrates A and B were left un-encapsulated and their surfaces exposed to ambient air to act as a reference in comparison with those that were encapsulated with either MoO_3_ or V_2_O_5_. Substrates C – G were encapsulated with 100 nm MoO_3_, while samples H – L were encapsulated with 100 nm V_2_O_5_. An oxide film thickness of 100 nm was chosen for this study as it provides continuity with previously reported work for these materials^21,22^. Use of relatively thick films should also importantly act to suppress the impact of environmental factors by separating the exposed oxide surface from the oxide:diamond interface, thus further maximising doping stability. It should also be noted that the transfer doping effect has been previously reported to saturate above a thickness of >2 nm for these oxide films^21,22^. Therefore, using a thickness of 100 nm should not adversely affect the efficiency of the doping process. Use of such “thick” films also ensure the ability to accurately perform X-ray Diffraction (XRD) measurements, which as will be shown, reveals some important structural changes in these oxide materials after exposure to elevated temperatures. Both MoO_3_ and V_2_O_5_ films were deposited by thermal evaporation in a vacuum of 2 × 10^–6^ mbar and their thickness controlled using a quartz crystal monitor during deposition. In previous work^21–24^, an *in-situ* annealing step immediately prior to oxide deposition was used to ensure desorption of any residual adsorbed material on the diamond surface.

**Table 1:** Summary of samples used in this work, grouped by surface acceptor.

Surface Acceptor	Substrate	Figure	Experiment
Air	A	2, 5	Stability with time and high temperature measurements in air
	B	5	High temperature measurements in vacuum
MoO_3_ 100 nm	C	3, 4	Stability with time (no *in-situ* anneal)
	D	3, 4	Stability with time (annealed *in-situ* at 400 °C prior to deposition)
	E	6, 7	High temperature measurements in air
	F	6, 7	High temperature measurements in vacuum
	G	6, 7	High temperature measurements in air encapsulated with HSQ
V_2_O_5_ 100 nm	H	3, 4	Stability with time (no *in-situ* anneal)
	I	3, 4	Stability with time (annealed *in-situ* at 400 °C prior to deposition)
	J	8, 9	High temperature measurements in air
	K	8, 9	High temperature measurements in vacuum
	L	8, 9	High temperature measurements in air encapsulated with HSQ

To investigate the potential impact of residual adsorbates on the diamond surface following oxide deposition, substrates C and H were encapsulated with 100 nm of MoO_3_ and V_2_O_5_ respectively with no *in-situ* anneal while substrates D and I received a 1-hour 400 °C *in-situ* anneal prior to encapsulation with 100 nm of MoO_3_ and V_2_O_5_ respectively. Substrates E and J both received a 400 °C *in-situ* anneal prior to oxide encapsulation and were then characterised by sheet resistance measurements in ambient atmosphere from room temperature to 300 °C. The same process was repeated for substrates F and K, however the high temperature measurements were performed in a low vacuum of 60 mTorr. Finally, substrates G and L were encapsulated with both MoO_3_ and V_2_O_5_ respectively after an *in-situ* 400 °C anneal and then both oxide layers were encapsulated with a spun layer of hydrogen silsesquioxane (HSQ). The HSQ was spun at a speed of 2000 rpm and baked on a hot plate for 2 minutes at 80 °C resulting in a ~600 nm thick layer. Oxide:HSQ encapsulated substrates G and L were measured from room temperature up to 300 °C in ambient atmosphere. HSQ was chosen as an encapsulation material due to the relatively minimal associated processing required for deposition *i.e*. to avoid potential modification to the oxide surface and associated degradation that may have resulted from exposure to additional chemicals, plasma, electron beam and high temperatures. HSQ is also robust at temperatures above 300 °C, making it suitable for the higher temperature measurements performed here^28^. All Hall measurements were performed using a Nanometrics HL5500PC Hall effect measurement system. Measurement of a calibration sample of known resistance, manufactured by Nanometrics, was used to ensure that any variation observed in the measurements taken were not attributed to instrument related error. High temperature sheet resistance measurements were performed by the four-probe method using a heated stage equipped with low vacuum capability. The structure and chemistry of the deposited oxide layers were inspected by X-ray diffraction after exposure to temperatures up to 300 °C as part of the elevated temperature measurement process. XRD measurements were performed using a Panalytical XPert-pro diffractometer (CuKα radiation corresponding to λ = 1.54178 Å wavelength), operating in a Bragg-Bretano reflection geometry. To achieve improved statistics and to probe the preferred film orientations, the sample was mounted on Panalytical zero background holder to allow the spinning of the sample at 60 rpm.

## Results and Discussion

### Inspection of stability with time

**Figure 2:**
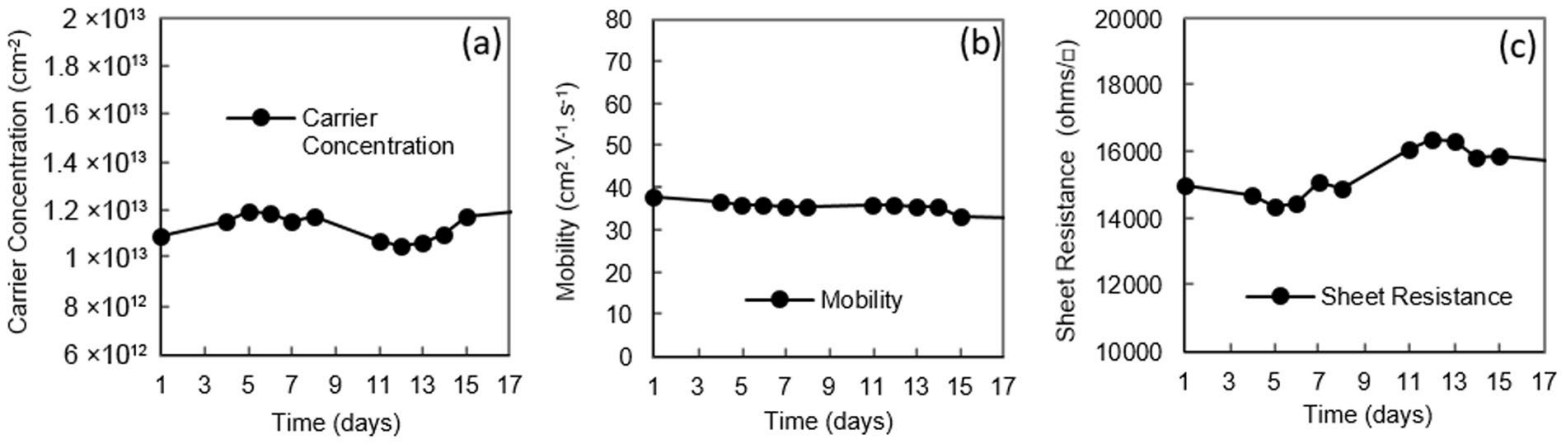
Hall measurements over time for an air-exposed (Sample A) H-diamond sample.

**Figure 3:**
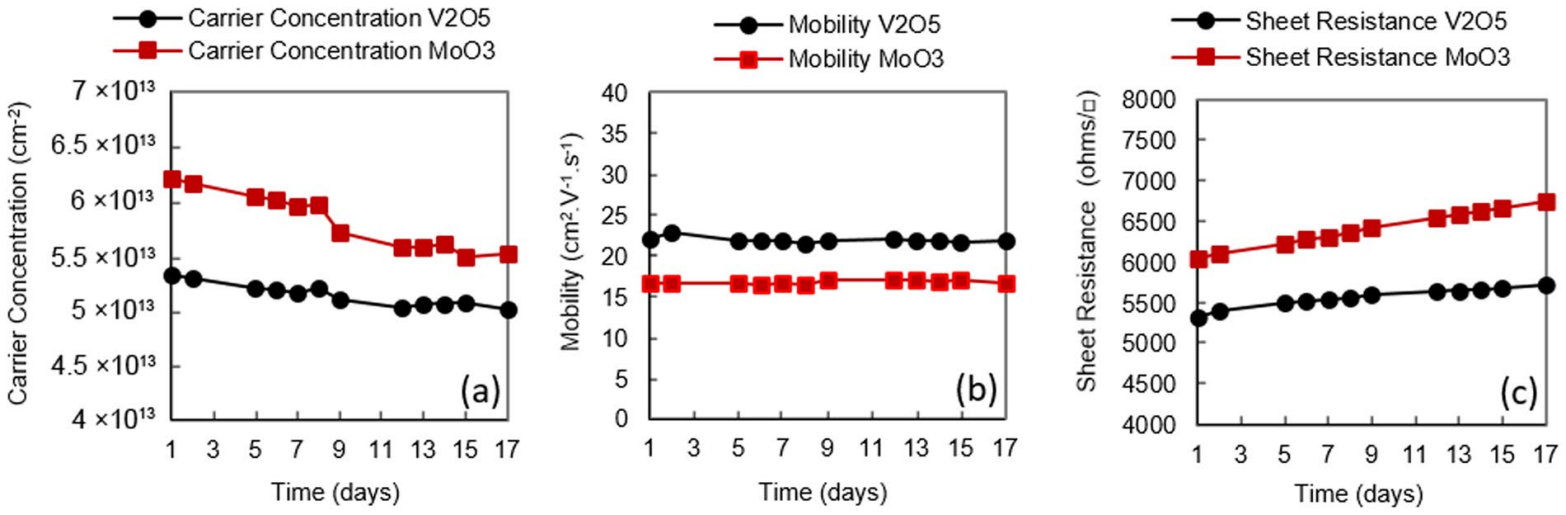
Hall measurements over time for MoO_3_ (Sample C) and V_2_O_5_ (Sample H) doped H-diamond samples with no *in-situ* annealing prior to oxide deposition.

**Figure 4:**
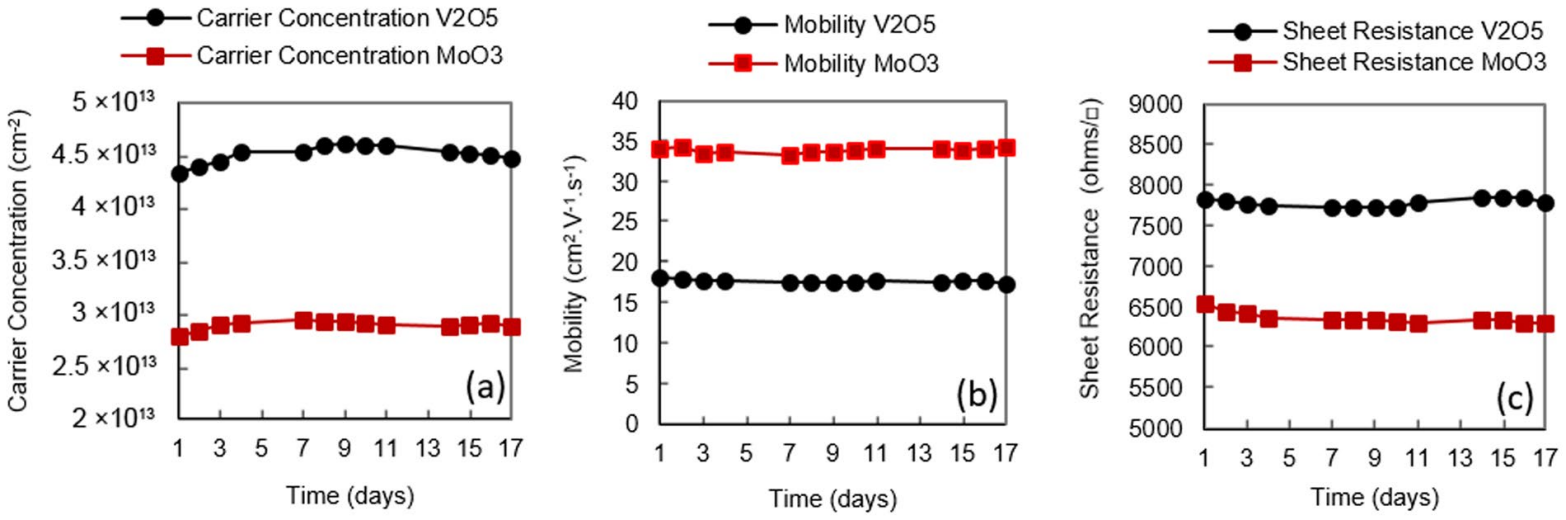
Hall measurements over time for MoO_3_ (Sample D) and V_2_O_5_ (Sample I) doped H-diamond samples with a 400 °C *in-situ* anneal prior to deposition.

Ambient air has been traditionally used as a surface acceptor medium for H-diamond^1,9^. While the poor thermal stability of air-induced transfer doping is well documented^9^, little work is reported on the general stability of air doping over extended periods of time. To investigate this, Substrate A was exposed to air after hydrogen termination and characterised by Hall measurement over a period of 17 days as shown in Fig. 2. During this period, the substrate was stored and measured within a class 10,000 clean room with temperature 21.6–22.2 °C and humidity of 45%+/−1% RH to provide as consistent a measurement environment as possible. Despite these measures, the hole carrier concentration was seen to fluctuate around an average of 1.1 × 10^13^ cm^−2^ with a deviation of 4.7% over the inspected time period. Sheet resistance showed a similar deviation of ~4.8% and a small degradation in hole mobility was observed after the 17-day period. This observed variation in conductivity is most likely attributed to minor environment-induced changes in the composition and/or concentration of air-borne acceptor species on the H-diamond surface, perhaps associated with small variations in humidity and temperature within the ranges measured. While sensitivity of transfer doping in H-diamond is an interesting topic in itself, further investigation into the origin of this instability was not undertaken as it is beyond the scope of this work; this data is included merely to illustrate the baseline stability of air-exposed H-diamond with time in our measurement environment. Substrates C and H were prepared in the same manner to Substrate A, but encapsulated with 100 nm of MoO_3_ and V_2_O_5_ respectively. To better understand the importance of performing a 400 °C *in-situ* anneal prior to oxide deposition, both oxide layers were deposited at room temperature with *no* thermal treatment performed prior to encapsulation. Similar to Substrate A, carrier concentration, mobility and sheet resistance were then monitored for 17 days for both Substrates C and H (Fig. 3). Storage and measurements of these substrates was also identical to that for Substrate A. Despite the lack of the *in-situ* anneal stage to ensure removal of any residual surface contaminants, both substrates demonstrated enhanced carrier concentration and reduced sheet resistance following deposition of MoO_3_ and V_2_O_5_, as is consistent with previously reported work^21,22^. In this instance, a lower sheet resistance was achieved with substrate H (V_2_O_5_) compared to substrate C (MoO_3_). The difference in overall conductivity for both the MoO_3_ and V_2_O_5_ encapsulated substrates shown here however is not necessarily indicative of the doping potential of each oxide, which may be affected by potential variation in substrate quality and hydrogen coverage. In comparison with the variation in carrier concentration and mobility observed with Substrate A, a steady decrease in carrier concentration and associated increase in sheet resistance was observed with time for both C and H. A slightly accelerated rate of degradation was observed however with MoO_3_ encapsulation (Substrate C) in comparison with V_2_O_5_ (Substrate H). After 17 days, the carrier concentration was observed to decrease by ~17% in Substrate C, which resulted in a sheet resistance increase of ~22%. This can be compared with Substrate H in which carrier concentration was found to decrease by ~15% and sheet resistance increase by ~11% over the same period. Interestingly, carrier mobility appeared to remain largely stable for both Sample C and H over this time period. The origin of the degradation observed with time for both MoO_3_ and V_2_O_5_ encapsulated H-diamond may be attributed to sensitivity of the oxide layers when exposed to atmosphere; MoO_3_ has been shown to be highly sensitive to air exposure (in particular to water and oxygen) resulting in a decrease of work function (WF) due to partial reduction at the oxide surface^29^. This decrease in WF is consistent with the observed reduction in carrier concentration here, as the doping performance of the surface material depends upon its respective electron affinity (Fig. 1). Arun Kuruvila *et al*. performed a similar study whereby surface transfer doping of graphene using 5 nm thick layers of V_2_O_5_ and MoO_3_ were inspected for the fabrication of light emitting diodes^30^. Their findings show similar degradation in surface transfer doping of MoO_3_ and V_2_O_5_ after exposure to air and note a greater stability of V_2_O_5_ compared to MoO_3_, also observed here. Their work also showed prolonged air exposure of MoO_3_ can lead to a reduced WF of 5.1 eV, a significant reduction from 6.9 eV. Therefore, the degradation in surface transfer doping over time observed for these substrates is believed to be at least partly attributed to exposure of the oxide layers to atmosphere. Substrates D and I were then prepared with 100 nm MoO_3_ and 100 nm V_2_O_5_ respectively. To inspect the impact of performing the surface ‘de-sorption’ stage as reported in previous work, both substrates received an *in-situ* anneal of 400 °C for 60 minutes prior to oxide deposition. Hall measurements were performed over a 17-day time period in the same cleanroom environmental conditions as the previously inspected substrates (Fig. 4). In contrast to Substrates C and H, which received no *in-situ* anneal, sheet resistance for Substrate D (MoO_3_) was observed to deviate by just 1.1% over the period measured. Sample I (V_2_O_5_) also exhibited a small deviation in sheet resistance of 0.6%. These results show a significant improvement in the stability of surface transfer doping with time for the diamond:oxide system resulting from the use of a 400 °C *in-situ* anneal prior to oxide deposition. The difference in mobility observed for samples D and I follows a consistent trend seen with transfer-doped hydrogen-terminated diamond, in which higher carrier density results in a reduction in carrier mobility^9,13,21–24^. While this extensively documented phenomenon is as yet not well understood, it likely results from increased coulomb and/or interface roughness scattering, as increased hole concentration within the diamond reduces proximity between the 2DHG and charge at the surface diamond:oxide interface. However, the resultant doping seen here is also substantially more consistent with time than that observed for the air-exposed H-diamond substrate, A. These results suggest that degradation in the transfer doping mechanism observed in Substrates C and H is related to encapuslation of residual adsorbed molecules under the oxide layers rather than exposure of their outer surface to atmosphere. However, given the reported atmospheric sensitivity of these oxide films, their encapsulation (with, for example, inert dielectric layers) may be essential to ensure longer term stability, particlarly for films thinner than 100 nm. This remains a point of investigation for future work.

### Inspection of stability at elevated temperatures

#### Air-exposed H-diamond

**Figure 5:**
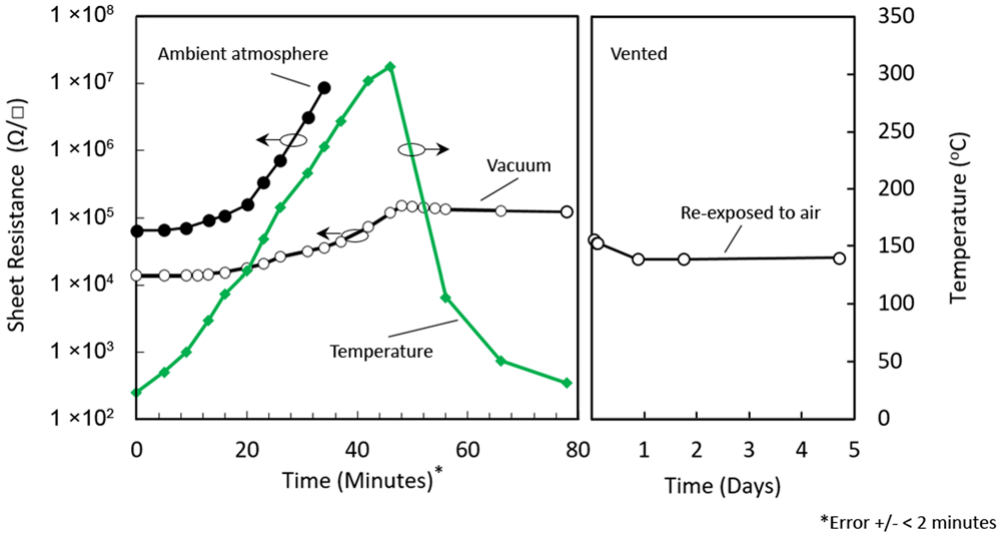
Hall measurements up to 300 °C for un-encapsulated samples (A and B) with cool down and vent.

To inspect the impact of elevated temperature on air-exposed H-diamond, sheet resistance measurements were performed on Substrates A and B from room temperature to 300 °C (Fig. 5). Measurements are presented on a single plot to allow ease of comparison and are therefore fitted to a single temperature profile resulting in a slight time error of +/− <2 minutes for each data point. For the air-doped Substrate A, increasing the temperature above 200 °C in ambient atmosphere caused the sheet resistance to increase to above 1 × 10^7^ Ω/□, beyond which the substrate conductivity became unmeasurable by our measurement system. Repeat measurements performed after Substrate A was cooled back down to room temperature indicated the substrate permanently retained this unmeasurably high sheet resistance. Substrate B was characterised in a similar fashion to Substrate A, but measured in a low vacuum of 60 mTorr (rather than ambient air) from room temperature to 300 °C. A much ‘slower’ degradation in sheet resistance was observed with increased temperature for Substrate B as attributed to the vacuum measurement environment. In contrast with Substrate A, Substrate B retained a decreased but measurable level of conductivity at 300 °C. The substrate was then cooled back down from 300 °C to room temperature in the same low-vacuum environment while measurements of sheet resistance were periodically taken during the cooling process. As shown in Fig. 5, little change in the sheet resistance was observed during this cool-down process. The measurement system was then vented once the substrate had reached room temperature and the sheet resistance measured periodically for 5 days with the substrate exposed once again to ambient air. An immediate and notable reduction in sheet resistance (~50%) was observed in Substrate B following re-exposure to air. This decrease continued for ~21 hours following re-exposure of the substrate to air before saturating and then following a similar trend to that shown in Fig. 2c for the remainder of the measurement period.

Surface transfer doping in H-diamond using ambient air as a surface acceptor is well known to exhibit poor thermal stability. Previous work has demonstrated that annealing H-diamond in high vacuum at ~ 400 °C removes any conductivity that is attributed to previous exposure to air^9^. In this work it was also found that the conductivity in the H-diamond returned after it was once again exposed to ambient air at room temperature. The recovery in conductivity observed following re-exposure to air indicated that the reduction in conductivity observed at 400 °C under vacuum was attributed to desorption of atmospheric species from the H-diamond surface rather than removal of hydrogen. Experiments at higher temperatures in this work indicated that a temperature of at least 700 °C is required for desorption of the chemisorbed hydrogen in high vacuum, Conversely, H-diamond was found to begin to lose hydrogen from the surface when heated in ambient air at much lower temperatures closer to ~200 °C^9^, due to oxidation of the surface. These findings agree well with the results achieved here for Substrates A and B, whereby the degradation in conductivity observed at elevated temperatures is due to a combination of partial oxidation of the diamond surface as well as desorption of adsorbed atmospheric species. For Substrate A, oxidisation of (and hence partial removal of hydrogen from) the diamond surface most likely resulted from heating of the substrate up to 300 °C in ambient air. Therefore, the conductivity did not recover when the substrate was re-exposed to ambient air. Conversely for Substrate B, the degradation in conductivity observed up to 300 °C was greatly reduced in comparison with Substrate A, and recovered substantially if not completely when re-exposed to ambient air. These results suggest that the surface hydrogen coverage in Substrate B remained mostly intact during heating in low vacuum and the observed increase in sheet resistance was predominantly attributed to partial removal of surface acceptor species during heating. Overall, these results emphasize once again the limited thermal stability associated with air-doped H-diamond in both vacuum and atmosphere.

### MoO_3_ encapsulated H-diamond

**Figure 6:**
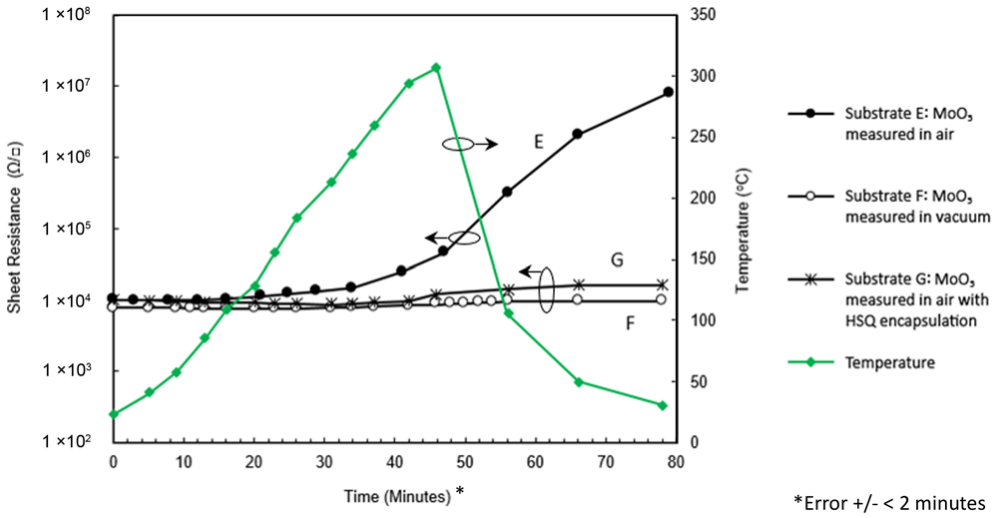
Sheet resistance measurements up to 300 °C for MoO_3_ encapsulated substrates, (E, F and G) with cool-down period.

To evaluate the thermal stability of H-diamond encapsulated with MoO_3_, similar sheet resistance measurements were performed on Substrates E, F and G from room temperature to 300 °C as shown in Fig. 6. Similar to Substrates A and B, measurements are presented on a single plot to allow ease of comparison and fitted to a single temperature profile. Substrate E was measured in ambient atmosphere and F in ‘low’ vacuum at a pressure of 60 mTorr. Substrate G, which included a 600 nm encapsulating layer of HSQ on top of the MoO_3_, was also measured in ambient air. Each of these substrates prior to oxide deposition were annealed *in-situ* at 400 °C for one hour to ensure maximum stability of the resultant transfer doping. Hall measurements taken for substrate G before and after encapsulation with HSQ showed no notable impact on surface conductivity with <1% change in sheet resistance. The results show conductivity in the MoO_3_ doped substrate E to be largely stable up to ~200 °C with a gradual increase in sheet resistance at higher temperatures. By contrast, Substrate F measured in low vacuum remains largely stable up to 300 °C with only a 4% increase in sheet resistance observed. Substrate G, which included a 600 nm HSQ encapsulation layer on top of the MoO_3_, showed greatly improved thermal stability when measured in air compared to Substrate E which was identical but without the HSQ encapsulation layer. During the cool down period from ~300 °C to room temperature, measurements performed on Substrate E demonstrated a continued increase in sheet resistance and a final value of close to 10 MΩ/□ measured at room temperature. While the diamond itself is unlikely to be modified by a temperature of 300 °C during measurements, the surface hydrogen termination and/or oxide stoichiometry is possibly more sensitive. In contrast, Substrate F (measured in low vacuum) showed a slight increase in sheet resistance after cooling down. The MoO_3_:HSQ encapsulated Substrate G measured in atmosphere showed more pronounced degradation than Substrate F after cooling down with sheet resistance roughly doubled. However, the effect is significantly reduced compared to that of the un-encapsulated air exposed MoO_3_ Substrate E. These results indicate that isolation of the MoO_3_ and/or diamond surface from atmosphere either through operation in vacuum or through hermetic encapsulation is essential to maximise the thermal stability of MoO_3_ transfer-doped H-diamond.

**Figure 7:**
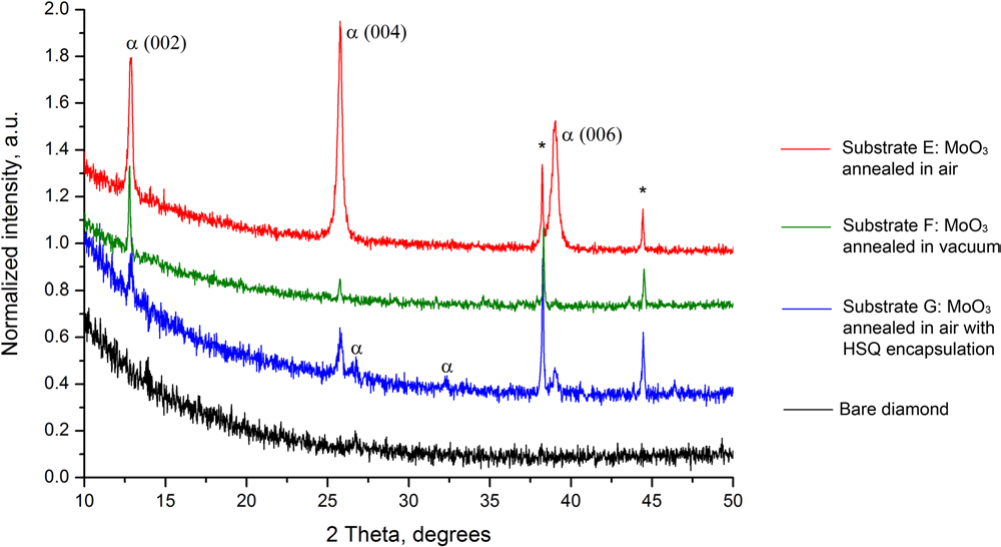
X-ray diffraction patterns of MoO_3_ films after Hall measurements. The reflections marked with asterisks originate from the silver contacts on the sample corners and α (G) corresponds to reflections from (012) and (110) peaks of α-MoO_3_.

X-ray diffraction studies were carried out on substrates E, F and G to identify any structural changes in the MoO_3_ film structure associated with heating of the substrates up to 300 °C in both ambient air and low vacuum. All diffraction experiments were carried out *ex-situ* after the high temperature sheet resistance measurements were completed. Figure 7 shows the normalised diffraction pattern for MoO_3_ coated H-diamond Substrates E, F and G. As a base line comparison, diffraction measurement of a bare un-encapsulated diamond substrate is also plotted. The reflections marked with asterisks originate from the silver contacts and were used as internal standard for normalisation of the intensity of the evaluated samples. There was a significant difference in the intensity of MoO_3_ peaks between substrates, indicating that the crystallinity of the films changes depending on the conditions during high temperature measurements. For Substrate E (measured in air), only 00*l* reflections associated with an orthorhombic MoO_3_ α-phase^31^ were observed, indicating that the MoO_3_ film was highly oriented along *c*-axis of the film. Similarly, the diffraction pattern of Substrate F (measured in low vacuum) displayed only 00*l* reflections of α-phase suggesting a strongly oriented film. However, the intensity of the reflections is significantly lower than compared with Substrate E which is indicative of lower crystallinity. Protecting MoO_3_ with HSQ still yields a more crystalline film although lacking strong preferred orientation as evident by additional reflections which are still attributed to the α-phase. These results show a temperature induced change in crystallinity of 100 nm MoO_3_ films on H-diamond up to 300 °C, however no clear correlation between surface conductivity of the H-diamond surface and crystallinity of the MoO_3_ is observed.

### V_2_O_5_ encapsulated H-diamond

**Figure 8:**
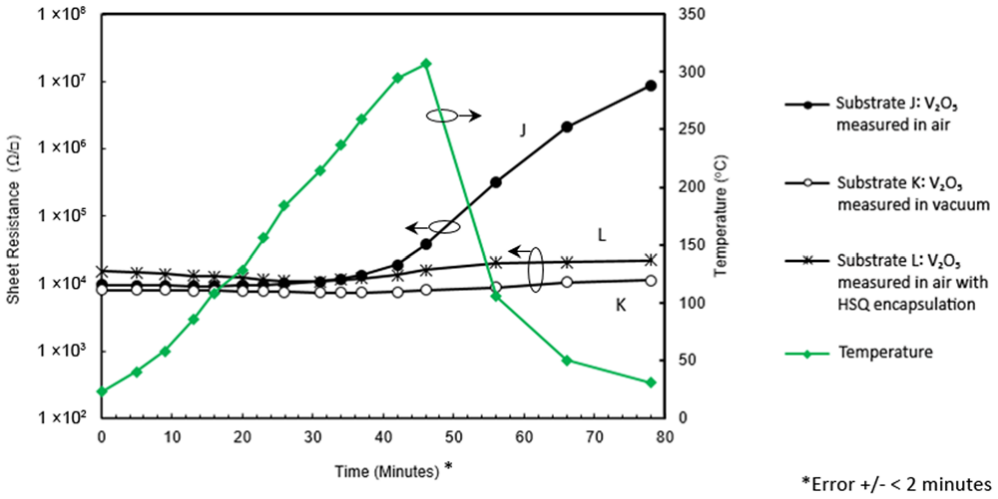
Sheet resistance measurements up to 300 °C for V_2_O_5_ encapsulated Substrates, (J, K and L) with cool-down period.

A similar set of experiments to those undertaken for MoO_3_ was performed to examine the thermal stability and structural integrity of V_2_O_5_-encapsulated H-diamond. Figure 8 shows the data collected from high temperature sheet resistance measurements performed up to 300 °C for three V_2_O_5_ encapsulated substrates: J (measured in ambient air), K (measured in a low vacuum of 60 mTorr) and L (measured in ambient air with a 600 nm layer of HSQ encapsulating the V_2_O_5_ film). These substrates all received a 400 °C *in-situ* anneal prior to oxide deposition to ensure maximum stability during these experiments. Hall measurements taken for substrate L before and after encapsulation with HSQ showed no notable impact on surface conductivity with <1% change in sheet resistance. Similar to the MoO_3_-encapsulated Substrate E, which was measured in air, sheet resistance of the V_2_O_5_-doped Substrate J was found to be relatively stable up to ~250 °C, but then increased at temperatures nearing 300 °C. In comparison, the V_2_O_5_ Substrate K measured in vacuum remained substantially more stable up to 300 °C akin to MoO_3_, with a relatively small increase in sheet resistance measured following cool down of the substrate back to room temperature. Substrate L, measured in air with V_2_O_5_:HSQ encapsulation, showed equivalent stability in sheet resistance as Substrate K up to ~ 300 °C. After returning to room temperature, Substrate J did not recover any of its lost surface conductivity. This behaviour is very similar to that observed for the MoO_3-_coated Substrate E, suggesting certain permanent changes at the diamond surface/interface may be the common factor in the observed conductivity loss at high temperatures in air. The V_2_O_5_-encapsulated substrate K measured in vacuum showed greatly improved stability by comparison. Substrate L with V_2_O_5_:HSQ encapsulation measured in ambient air up to 300 °C also showed similar degradation to that observed for MoO_3_:HSQ (Substrate G). These results suggest that optimised encapsulation of these oxide films with a more robust dielectric material should reduce conductivity loss even further at temperatures up to 300 °C.

**Figure 9:**
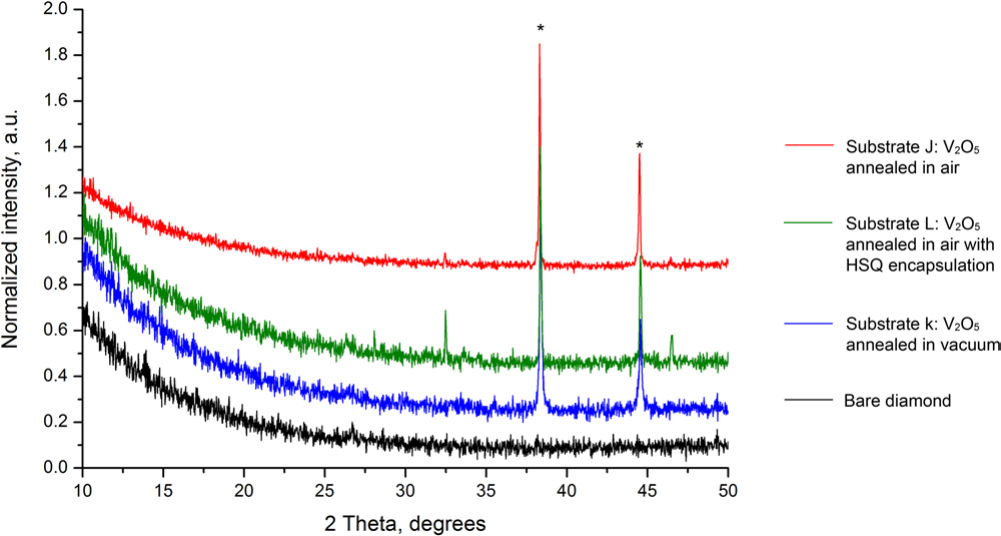
X-ray diffraction patterns of V_2_O_5_ films after Hall measurements. The reflections marked with asterisks originate from the silver contacts on the sample corners. The peaks at ca. 32.5° and 46.6° are unidentified. However, careful assessment against JCPDF database has shown they don’t belong to any of V or Ag oxides.

Similar to the trials using MoO_3_, XRD measurements were performed on substrates J, K and L to inspect any thermally induced structural change to the V_2_O_5_ films (Fig. 9). In contrast to the XRD measurements performed on the MoO_3_ films, only reflections associated with the silver contacts are visible in these results. Therefore, the V_2_O_5_ films appeared to remain amorphous after heating to 300 °C in air, vacuum and with HSQ encapsulation in air. V_2_O_5_ would therefore appear to be substantially less structurally sensitive to the effects of elevated temperatures in both vacuum and atmosphere when compared with MoO_3_ films of the same thickness. These XRD results also suggest there is little correlation between any thermally-induced physical modification to both the MoO_3_ and V_2_O_5_ layers and the electrical results achieved for both MoO_3_ and V_2_O_5_.

## Conclusions

Transition metal oxides such as MoO_3_ and V_2_O_5_ have previously been demonstrated to improve stability and efficiency of surface transfer doping on hydrogen-terminated diamond as verified by both Hall measurement and photoemission spectroscopy^21,22^. In this work, the importance of performing an *in-situ* anneal prior to oxide deposition was verified to be essential to maximise stability of the resultant transfer doping over time. Further investigation into the optimisation of these anneal conditions, while not part of this work, may further enhance stability of these oxide transfer-doped systems and improve their integration potential into a device process flow. Inspection of the electrical operation of MoO_3_ and V_2_O_5_ transfer-doped H-diamond was performed by sheet resistance measurements from room temperature to 300 °C. This revealed sensitivity to ambient atmosphere at elevated temperatures, resulting in a lasting loss in conductivity of the diamond substrate. This may be due to reduction in electron affinity of the oxide layer while exposed to atmosphere at high temperatures or potentially a modification of the diamond hydrogen termination. In both instances this would result in reduced transfer-doping of the diamond. High temperature measurements in vacuum and with encapsulation of the oxide film in air showed substantially improved stability. These results suggest isolation of the diamond:metal-oxide interface from ambient atmosphere allows for good stability at temperatures up to 300°C and a vast improvement in comparison with air-induced transfer doping. Stability over time when exposed to atmosphere is also significantly improved and further demonstrates the viability of transition metal oxides such as MoO_3_ and V_2_O_5_ on H-diamond for surface conducting electronic devices.

## References

[1] Wort CJH, Balmer RS (2008). Diamond as an Electronic Material. Materials Today.

[2] Hirama, K. *et al*. High-Performance P-Channel Diamond MOSFETs with Alumina Gate Insulator, *IEDM Technical Digest*, **873** (2007).

[3] Wang JJ (2016). Comparison of Field-Effect Transistors on Polycrystalline and Single-Crystal Diamonds. Diamond & Related Materials.

[4] Kasu, M. & Oishi, T. Recent Progress of Diamond Devices for RF Applications, *IEEE Compound Semiconductor Integrated Circuit Symposium* (2016).

[5] Mainwood A (2006). Theoretical modelling of dopants in diamond. Journal of Materials Science: Materials in Electronics.

[6] Kalish, R. Doping of diamond, *Carbon* **37** (1998).

[7] Yokoya, T. *et al*. Origin of the Metallic Properties of Heavily Boron-doped Superconducting Diamond, *Nature* **438** (2005). 10.1038/nature04278 16319887

[8] Chicot, G. *et al*. Hole Transport in Boron Delta-Doped Diamond Structures, *Applied Physics Letters* **101**, (2012).

[9] Maier F, Riedel M, Mantel B, Ristein J, Ley L (2000). Origin of Surface Conductivity in Diamond. Physical Review Letters.

[10] Kasu M (2007). Power Transistors: Fundamentals and Applications. Diamond & Related Materials.

[11] Russell SAO, Sharabi S, Tallaire A, Moran DAJ (2012). Hydrogen-Terminated Diamond Field-Effect Transistors with Cutoff Frequency of 53 GHz. IEEE Electron Device Letters.

[12] Ueda K (2006). Diamond FET using High-Quality Polycrystalline Diamond with f/sub T/ of 45 GHz and f/sub max/ of 120 GHz. IEEE Electron Device Letters.

[13] Chen W, Qi D, Gao X, Wee ATS (2009). Surface Transfer Doping of Semiconductors. Progress in Surface Science.

[14] Strobel P, Riedel M, Ristein J, Ley L (2004). Surface Transfer Doping of Diamond. Nature.

[15] Edmonds MT (2012). Surface Transfer Doping of Hydrogen-Terminated Diamond by C60F48: Energy Level Scheme and Doping Efficiency. Journal of Chemical Physics.

[16] Qi D (2007). Surface Transfer Doping of Diamond (100) by Tetrafluoro-tetracyanoquinodimethane. Journal of the American Chemical Society.

[17] Strobel P (2006). Surface Conductivity Induced by Fullerenes on Diamond: Passivation and Thermal Stability. Diamond & Related Materials.

[18] Daicho A, Saito T, Kurihara S, Hiraiwa A, Kawarada H (2014). High-Reliability Passivation of Hydrogen-Terminated Diamond Surface by Atomic Layer Deposition of Al2O3. Journal of Applied Physics.

[19] Hirama K, Sato H, Harada Y, Yamamoto H, Kasu M (2012). Thermally Stable Operation of H-Terminated Diamond FETs by NO2 Adsorption and Al2O3 Passivation. IEEE Electron Device Letters.

[20] Liu JW, Liao MY, Imura M, Koide Y (2012). Band offsets of Al_2_O_3_ and HfO_2_ Oxides Deposited by Atomic Layer Deposition Technique on Hydrogenated Diamond. Applied Physics Letters.

[21] Crawford KG (2016). Enhanced surface Transfer Doping of Diamond by V_2_O_5_ with Improved Thermal Stability. Applied Physics Letters.

[22] Russell SAO (2013). Surface Transfer Doping of Diamond by MoO_3_: A Combined Spectroscopic and Hall Measurement Study. Applied Physics Letters.

[23] Tordjman M, Saguy C, Bolker A, Kalish R (2014). Superior Surface Transfer Doping of Diamond with MoO_3_. Advanced Material Interfaces.

[24] Verona C (2016). Comparative Investigation of Surface Transfer Doping of Hydrogen Terminated Diamond by High Electron Affinity Insulators. Journal of Applied Physics.

[25] Verona C (2016). V_2_O_5_ MISFETs on H-Terminated Diamond. IEEE Transactions on Electron Devices.

[26] Vardi A, Tordjman M, del Alamo JA, Kalish R (2013). A Diamond:H/MoO_3_ MOSFET. IEEE Electron Device Letters.

[27] Rossi MC, Spaziani F, Salvatori S, Conte G (2003). Electronic Properties of Hydrogen and Oxygen Terminated Surfaces of Polycrystalline Diamond Films. Phys. Status Solidi.

[28] Häffner M (2007). Influence of temperature on HSQ electron-beam lithography. Journal of Vacuum Science & Technology B, Nanotechnology and Microelectronics: Materials, Processing, Measurement, and Phenomena.

[29] Irfan I (2010). Energy Level Evolution of Molybdenum Trioxide Interlayer between Indium Tin Oxide and Organic Semiconductor. Applied Physics Letters.

[30] Kuruvila A (2014). Organic Light Emitting Diodes with Environmentally and Thermally Stable Doped Graphene Electrodes. Journal of Materials Chemistry C.

[31] Chang W (2011). Post-Deposition Annealing Control of Phase and Texture for the Sputtered MoO_3_ Films. CrystEngComm.

